# P-279. Longitudinal Trends in ART and HIV Drug Resistance Mutations Based on HIV Diagnosis Era: Findings from an Urban Clinic in Pittsburgh

**DOI:** 10.1093/ofid/ofaf695.500

**Published:** 2026-01-11

**Authors:** Rafael Garcia Sturgill, Cassandra Oehler, Chiu-bin hsiao

**Affiliations:** Allegheny Health Network, Pittsburgh, Pennsylvania; Allegheny General Hospital, Positive Health Clinic, Center for Inclusion Health, AHN, Drexel University College of Medicine, Pittsburgh, Pennsylvania; Allegheny General Hospital, Positive Health clinic, Center for Inclusion Health, AHN;Drexel University, College of Medicine, Pittsburgh, Pennsylvania

## Abstract

**Background:**

ART development has improved quality of life and prolonged life expectancy of PWHIV to near that of the general population. Since 2006 ART regimens have become simpler, more effective, and easier to tolerate with a high barrier to develop HDRM. It is important to quantify the degree to which ART usage has impacted HDRM in ART-naive and treatment experienced populations over time.

**Figure:**
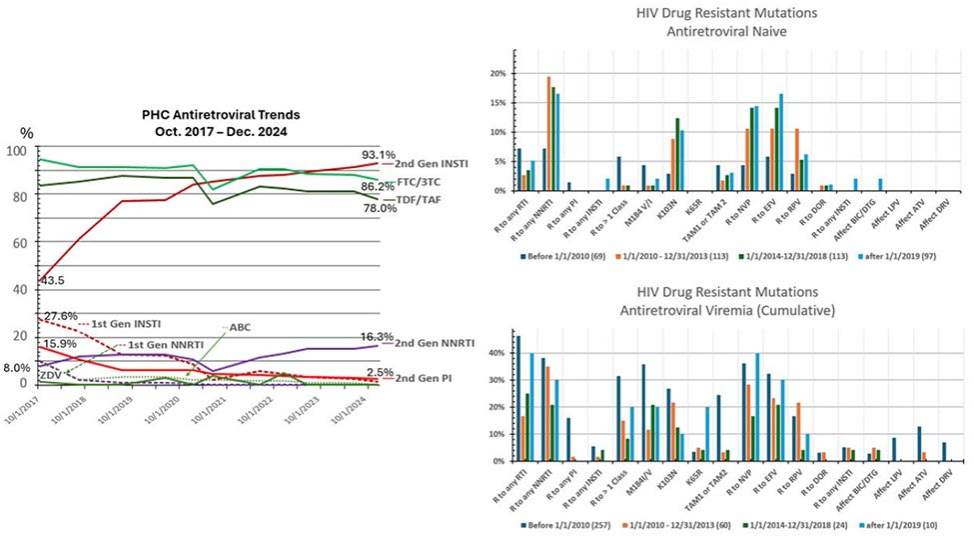
ART trend and HDRM testing

Left: Trends of ART drugs from 2017 until 2024:

Right Top: HDRM testing of treatment naïve patients by period of HIV diagnosis

Right Bottom: HDRM testing of treatment experienced patients by period of HIV diagnosis

**Methods:**

All reports on every HIV drug resistance mutation (HDRM) testing and ART were reviewed from 2017 –2024 in a bi-annual fashion. HDRM were classified in four periods based on HIV diagnosis year (A: prior to 2010, B: 2010-2014, C: 2015-2018, D: after 2019). Stanford HIV Database was used to determine the impact of HDRM findings.

**Results:**

Among 1057 patient records, 743 patients (392 naive, 351 experienced) had reports of HDRM. From 2017-2024, second generation INSTI-based regimens increased from 43.5% to 93.1%, use of first generation INSTI-based regimens decreased from 27.6% to 0.2%. PI-based decreased from 15.9% to 2.5%. NNRTI regimens increased from 8.0% to 15%, mainly RPV based. TAF or TDF/FTC backbone was used in about 75% of the regimens. Despite the shift of ART regimens to mostly second generation INSTI-based regimens there has been no increase in HDRM to INSTI in experienced patients in period C and D. HDRM impact on NNRTI was stable at 10-20%: for both groups, about half (5-10%) had RPV HDRM, see figure. Two cases (period D) were infected with HIV strain that carried INSTI HDRM with G140S and Q148 codon. One (period A) developed new INSTI HDRM (E92Q, T97A, E138T, S147G, N155H) and NNRTI HDRM (K101P, K103N) while on DTG/RPV regimen. Despite multiple viremic events noted while on second generation INSTI or DRV based regimens no patient developed new INSTI or PI HDRM.

**Conclusion:**

Patients on TAF (TDF)/ FTC/ DTG or BIC or DRV, DTG + DRV/c regimen were least likely to develop HDRM. Viremic events while on DTG/RPV may promote HDRM impacting second-generation INSTI (BIC or DTG). RPV HDRM in ART naive population limits the potential use of CAB/RPV LA regimen. No DRV HDRM in the treatment naive patients were noted. INSTI HDRM testing should be considered for newly diagnosed HIV, especially, we have observed INSTI HDRM in ART naive cases in last 2 years.

**Disclosures:**

Chiu-bin hsiao, MD, Viiv/GSK: Grant/Research Support

